# Increasing Access to Subsidized Artemisinin-based Combination Therapy through Accredited Drug Dispensing Outlets in Tanzania

**DOI:** 10.1186/1478-4505-9-22

**Published:** 2011-06-09

**Authors:** Edmund Rutta, Bryceson Kibassa, Brittany McKinnon, Jafary Liana, Romuald Mbwasi, Wilson Mlaki, Martha Embrey, Michael Gabra, Elizabeth Shekalaghe, Suleiman Kimatta, Hiiti Sillo

**Affiliations:** 1 Management Sciences for Health, Center for Pharmaceutical Services, Arlington, USA; 2 Tannzania Food and Drug Authority, Dar es Salaam, Tanzania; 3 McGill University, Department of Epidemiology, Biostatics' and Occupational Health, Montreal, Canada; 4 Management Sciences for Health, Center for Pharmaceutical Services, Dar es Salaam, Tanzania

## Abstract

**Background:**

In Tanzania, many people seek malaria treatment from retail drug sellers. The National Malaria Control Program identified the accredited drug dispensing outlet (ADDO) program as a private sector mechanism to supplement the distribution of subsidized artemisinin-based combination therapies (ACTs) from public facilities and increase access to the first-line antimalarial in rural and underserved areas. The ADDO program strengthens private sector pharmaceutical services by improving regulatory and supervisory support, dispenser training, and record keeping practices.

**Methods:**

The government's pilot program made subsidized ACTs available through ADDOs in 10 districts in the Morogoro and Ruvuma regions, covering about 2.9 million people. The program established a supply of subsidized ACTs, created a price system with a cost recovery plan, developed a plan to distribute the subsidized products to the ADDOs, trained dispensers, and strengthened the adverse drug reactions reporting system. As part of the evaluation, 448 ADDO dispensers brought their records to central locations for analysis, representing nearly 70% of ADDOs operating in the two regions. ADDO drug register data were available from July 2007-June 2008 for Morogoro and from July 2007-September 2008 for Ruvuma. This intervention was implemented from 2007-2008.

**Results:**

During the pilot, over 300,000 people received treatment for malaria at the 448 ADDOs. The percentage of ADDOs that dispensed at least one course of ACT rose from 26.2% during July-September 2007 to 72.6% during April-June 2008. The number of malaria patients treated with ACTs gradually increased after the start of the pilot, while the use of non-ACT antimalarials declined; ACTs went from 3% of all antimalarials sold in July 2007 to 26% in June 2008. District-specific data showed substantial variation among the districts in ACT uptake through ADDOs, ranging from ACTs representing 10% of all antimalarial sales in Kilombero to 47% in Morogoro Rural.

**Conclusions:**

The intervention increased access to affordable ACTs for underserved populations. Indications are that antimalarial monotherapies are being "crowded out" of the market. Importantly, the transition to ACTs has been accomplished in an environment where the safety and efficacy of the drugs and the quality of services are being monitored and regulated. This paper presents a description of the pilot program implementation, results of the program evaluation, and a discussion of the challenges and recommendations that will be used to guide rollout of subsidized ACT in ADDOs in the rest of Tanzania and possibly in other countries.

## Background

Private sector drug outlets, including drug shops, general shops, and market vendors, are major sources of medicines in sub-Saharan Africa. A review of studies from some countries found that around half of caregivers initially sought medicines for the treatment of common childhood illnesses from private drug sellers [1].

Historically, in Tanzania, retail drug shops commonly known as *duka la dawa baridi *(DLDB) are the most common source of medicines in the private sector [2]. Because almost no registered pharmacies can be found outside major urban centers, DLDB provide an essential service to rural and peri-urban populations who have limited access to registered pharmacies, and even act as a safety net when public facilities experience drug stock outs. The Tanzania Food and Drugs Authority (TFDA) authorize the estimated 6,800 DLDB to sell non-prescription drugs in the private sector [3]; however, the majority also illegally stock and dispense an array of prescription-only drugs [4]. Most DLDB have inadequate facilities for properly storing medicines and wide variation in the quality and prices of medicines available. Dispensers and owners typically lack qualifications and training, demonstrate poor adherence to TFDA regulations, and have little or no business knowledge. Moreover, regulation and supervision by inspectors is often inadequate [4,5].

In 2002, the Ministry of Health and Social Welfare and TFDA worked with Management Sciences for Health's (MSH) Strategies for Enhancing Access to Medicines Program, with funding from the Bill & Melinda Gates Foundation, to pilot an innovative private sector drug seller program to transform the DLDB into government-accredited drug dispensing outlets (ADDO)--*Duka la Dawa Muhimu *in Kiswahili. The goal of the ADDO program is to improve access to affordable, quality medicines and pharmaceutical services in rural and peri-urban areas where there are few or no registered pharmacies. The accreditation process for ADDOs takes a holistic approach, with a focus on training and changing behavior and expectations of those who use, own, regulate, and work in the drug shops.

**Figure 1:**
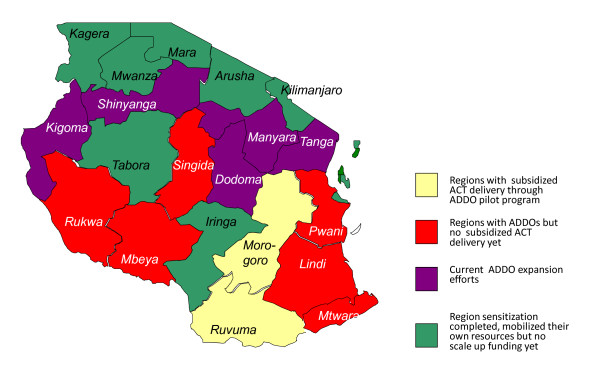
**Map of Tanzania showing status of ADDO expansion and subsidized ACT distribution (as of December 2008)**.

The accreditation process involving several key steps and different stakeholders at various levels has been described elsewhere [6]. The success of the ADDO pilot program in the Ruvuma region led to the Ministry of Health and Social Welfare's decision to expand the ADDO program to all 21 mainland regions of the country. To date, in addition to the pilot region, ADDOs are fully functional in seven other regions of Tanzania. Funding has come from the Government of Tanzania for rollout in Rukwa and Mtwara regions and from the U.S. Agency for International Development in Morogoro region. Other regions have received support from the Global Fund to Fight AIDS, Tuberculosis and Malaria and the Danish International Development Agency. In 2009, the government passed a regulation declaring that all DLDB be phased out by January 2011 (GN. No. 19 of 16^th ^January 2009) at which time ADDO coverage was expected to be complete (Figure 1).

Malaria is the biggest public health problem in Tanzania and is responsible for 30% of the national disease burden, 35% of hospitalizations, and 37% of deaths in children under five years [7]. Tanzania's 2005 malaria treatment policy changed first-line treatment for uncomplicated malaria from sulfadoxine-pyrimethamine (SP) to artemisinin-based combination therapy (ACT)--specifically artemether-lumefantrine (sometimes designated as ALu). The National Malaria Control Program (NMCP) subsequently identified the ADDO program as a potential private sector mechanism to supplement the distribution of subsidized ACTs in public facilities and increase access to the first-line antimalarial in rural and underserved areas [7]. NMCP also views the delivery of subsidized ACTs through ADDOs as perhaps the best approach to home-based management of malaria in Tanzania, because ADDOs can supply affordable and quality malaria medicines close to where they are needed and can sensitize communities to the need to seek early treatment and to adhere to the full course of treatment [8].

With funding from the Global Fund, in December 2006, Tanzania began providing ACTs through public and mission health facilities free of charge to all children under age five and at a subsidized price for the rest of the population [9]. However, a significant access problem remains for the approximately 35% of Tanzanians who seek treatment for malaria in the private sector [9]. Availability of ACT products in the private sector had generally been limited to registered pharmacies in urban areas where the price of a course of therapy is around 8-10 U.S. dollars (USD)--well beyond the reach of individuals living in rural and peri-urban communities who need them the most. This has left millions of Tanzanians in rural areas to rely on public sector facilities for access to the recommended first-line treatment for malaria, or if they seek treatment from more convenient retail outlets, to receive suboptimal therapies, primarily SP, which is more affordable and widely available. Distribution of subsidized ACTs through ADDOs is seen as the best way to expand the availability of ACTs through legally recognized and regulated outlets.

In 2007, with funding from the President's Malaria Initiative (PMI) and technical support from MSH's Rational Pharmaceutical Management Plus (RPM Plus) Program, TFDA and NMCP began a pilot program to make subsidized ACTs available through ADDOs in 10 districts in the Morogoro and Ruvuma regions. At that time, the *National Guidelines for Malaria Diagnosis and Treatment *identified ADDOs as a first-level category provider of ACT products [7]. The pilot program covered about 2.9 million people, an estimated 8.4% of the population of Tanzania. This intervention sought to capitalize on the ADDO program's improved systems, including regulatory and supervisory support, dispenser training, record keeping practices, and proper drug storage [5]. The pilot program established a supply of subsidized ACTs, created a price system with a cost recovery plan, developed a plan to distribute the subsidized products to the ADDOs, trained dispensers on treatment policy and record keeping related to ACT dispensing, and strengthened adverse drug reaction (ADR) reporting system. Approximately one year after initiation, MSH collaborated with TFDA to conduct the first comprehensive review of the pilot program.

The results of the review will provide government stakeholders with evidence needed to guide policies related to increasing access to affordable and effective antimalarials through the private sector. Specific policy and implementation issues include ACT packaging and pricing, supply logistics, regulatory oversight and supervision, training, monitoring drug safety, and behavior change and communication. In addition, the data on the subsidization of preferred treatment will provide policy makers with key information on the effectiveness of that approach.

## Methods

### Study area

The pilot program took place in five districts of the Morogoro region and five districts of Ruvuma region (Figure 1). The burden of malaria in both regions of the pilot program is high, with the prevalence of malaria in children age 6-59 months estimated at 15.7% in Morogoro and 23.9% in Ruvuma [10]. Ruvuma was the initial pilot region for the ADDO program, with the first ADDOs receiving accreditation in 2003. In Morogoro, the first shops received accreditation in 2006. In July 2007, ADDOs began supplying subsidized ACTs in Ulanga and Kilombero districts of Morogoro region and in the five districts of Ruvuma region. ACT distribution expanded to three more districts in Morogoro, Kilosa, Mvomero, and Morogoro Rural, in November 2007, Morogoro Urban was the only district in the two regions that was not included in the pilot because the ADDO accreditation program did not extend to urban areas pending a policy decision by the Ministry of Health and Social Welfare to allow ADDOs to operate near registered pharmacies.

### Policy changes

The policy and regulatory environment in Tanzania allowed the private sector to deliver subsidized ACTs. The NMCP had already identified the ADDO program as a potential mechanism to supplement public sector distribution of subsidized ACTs and increase access in rural and underserved areas [7]. To allow ADDOs to dispense ACTs, TFDA added ACT products to the existing limited list of prescription-only medicines ADDOs are legally authorized to dispense. RPM Plus also worked with TFDA to revise the ADDO dispenser's manual to reflect the malaria treatment policy change from SP to ACT. Finally, while artemisinin monotherapies have not been as widely used or available in Tanzania as they are in parts of Asia, the TFDA took steps to stop granting local production and importation permits for oral dosage forms of artemisinin-based monotherapies in August 2008. This action was prompted by concerns over emerging resistance to artemisinin in Southeast Asia and the importance of preserving the efficacy of artemisinin-based products as long as possible [11].

### Packaging and pricing ACTs for ADDOs

**Figure 2:**
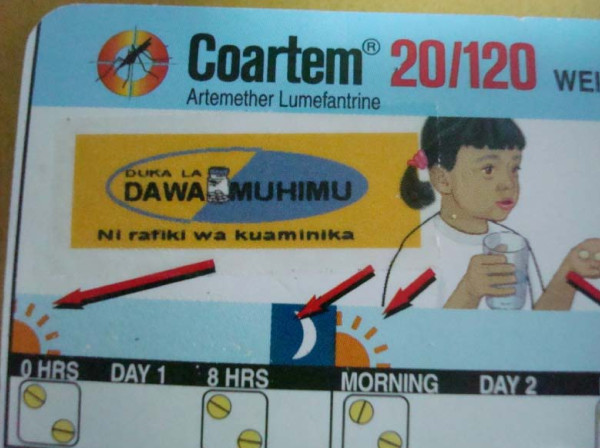
**ADDO sticker on ACT blister pack**.

Novartis chose to package its ADDO-dispensed product to look like that used for public sector facilities and to differentiate it from their commercial sector packaging of Coartem^®^. This decision allowed those visiting either ADDOs or public clinics to receive the same dosage instructions and public health messages; however, NMCP was concerned that using the exact same packaging might result in significant product diversion from public facilities to ADDOs. To reduce this likelihood, a tamper-proof sticker was used on subsidized packages sold in ADDOs, thereby, making it easier for TFDA inspectors to differentiate the two (Figure 2).

As in the public sector, ADDOs have four different-colored ACT blister packs that correspond to the different doses required according to body weight. To simplify instructions for the ADDOs, however, age categories were created that approximate the weight categories for each dose. Table 1 shows information on age, weight, dose, and price for each of the ACT packages available in ADDOs. All ADDOs received a laminated poster providing this information (in Kiswahili), which they are required to display in the shops.

**Table 1:** Information on ACTs available in ADDOs

Color of package	Age	Weight (kg)	Dose	Retail Price in Tanzanian shillings (TSH)/USD*
**Yellow**	**3 months-3 years**	**5-15**	**1 × 6**	**500/0.38**
**Blue**	**3-8 years**	**15-25**	**2 × 6**	**500/0.38**
**Red**	**8-12 years**	**25-35**	**3 × 6**	**1,500/1.15**
**Green**	**12 years and up**	**35+**	**4 × 6**	**1,500/1.15**

*Based on 2006 foreign exchange rate 1 USD = 1,300 TSH

In public health facilities, ACTs are free for children under five and cost TSH 300 per course of therapy (USD 0.23) for other patients. This cost excludes the cost for laboratory tests for malaria diagnosis, which when indicated, adds TSH 300-500 (USD 0.23-0.38) to the patient's cost. Anecdotal evidence suggests that facilities adhere to this price and provide free access to children under five. It was determined, however, that for this pilot, TSH 300 for the course of therapy was too low for ADDO owners to recover operational costs and produce a large enough profit margin to provide an incentive for them to stock the product. We examined drug shop clients' ability to pay based on the sales of other pharmaceuticals sold at ADDOs and reviewed research examining willingness and ability to pay for ACTs [12]. ADDO owners were consulted as part of the price negotiations, and they agreed to a fixed price for subsidized ACTs (prices for other pharmaceuticals in the ADDOs are not fixed). As a result of the analysis and negotiations, the final prices of subsidized ALu distributed through ADDOs were TSH 1,500 (USD 1.12) for an adult course of therapy and TSH 500 (USD 0.38) for a child's course of therapy.

### ACT supply logistics

To procure the supply of ACTs for the pilot program, PMI negotiated with Novartis, who agreed to supply the product to be distributed through ADDOs at the same price as the public sector receives. Procurement for ADDOs followed the same procedure as procurement in the public sector, where the consignee fills out and submits ACT order submission forms to the World Health Organization. For the pilot program, ACTs were procured in two consignments, which provided an opportunity to track the first consignment's sales before procuring the second consignment. In addition, because ACTs have a relatively short two-year shelf life, this ensured that the product in the second consignment would have close to the full useful life available. The program negotiated with the TFDA to obtain a waiver of the 2% importation fee that TFDA charges for all pharmaceutical imports.

To determine quantities of ACTs needed for ADDOs for the pilot program, we used the morbidity method of quantification because no historical data was available on ACT sales in ADDOs. Therefore, we reviewed dispensing registers from a sample of 78 ADDOs to determine the number and age of customers who sought antimalarial treatment during the preceding 12-month period in Ruvuma and Morogoro regions. Interviews with owners and dispensers of selected ADDOs provided additional information. We also reviewed the Southern Highland Pharmacy's database in Songea town, which has a comprehensive record of ADDOs in Ruvuma region that have purchased from them since 2003. Estimates took into account the transmission patterns of malaria in the two regions and the age-specific dosage categories for ACTs, which were based on the NMCP criteria for estimating ACT needs for public health facilities. RPM Plus estimated that 661,393 courses of treatment would be needed to supply the approximately 650 ADDOs in the target districts for the first year.

The plan to distribute ACTs to ADDOs was developed based on the existing private sector supply chain in Tanzania, where private pharmaceutical wholesalers are the main source of pharmaceuticals. Pyramid Pharma Ltd, based in Dar es Salaam, was chosen as the ACT distributor for ADDOs based on the following criteria: adequate and secure storage facility, readiness to track ACT inventory, reliable transportation to regional sub-distributors, high-quality record keeping, local availability of a wholesale unit in Ruvuma region, and previous working experience with major public and private sector clients. Pyramid Pharma was given responsibility to receive ACT consignments upon arrival in the country and to clear, store, and distribute the drugs to the two regional distributors.

**Figure 3:**
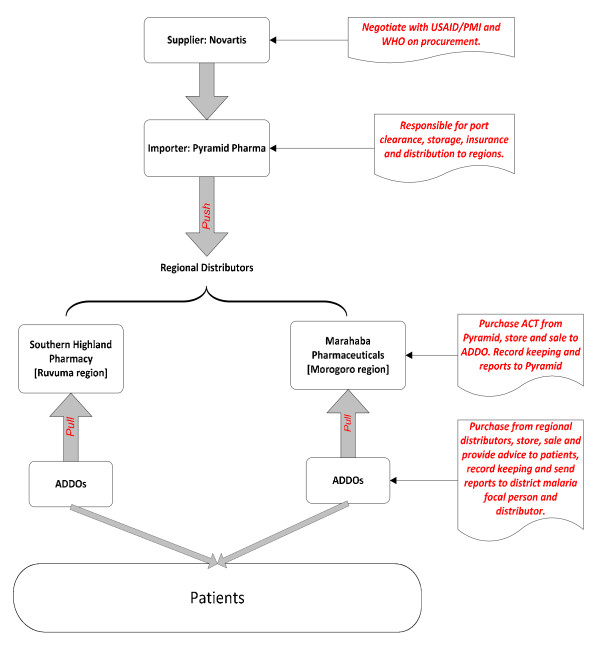
**Supply and distribution of subsidized ACT products through ADDOs**.

Pyramid Pharma identified Southern Highland Pharmacy as its subsidiary distributor for Ruvuma region and Marhaba Pharmaceuticals Ltd for Morogoro region. These regional distributors were already the main suppliers of ADDOs; 89% of all ADDOs in Ruvuma regularly procured their pharmaceutical supplies from Southern Highland pharmacy [13]. To ensure that the regional distributors correctly supply ACT to the intended ADDOs, buyers must present their ADDO accreditation certificate and sign the distributor's ACT register. The key feature of the supply system design was a combination of "push" and "pull." The national distributor agency (Pyramid Pharma) "pushes" predetermined quantities of ACTs to the regional distributors, Southern Highland Pharmacy and Marhaba Pharmaceuticals Ltd. ADDOs are expected to "pull" ACT quantities from the regional distributors based on their needs. This design maximizes private sector supply chain efficiency by avoiding excess ACT stock or stock outs because ADDOs can procure drugs based on their needs during their normal purchasing schedule. Figure 3 shows a breakdown of the chain of supply for ACTs in ADDOs.

### Regulatory oversight and supervision

Generally, the regulation of ACT distribution through ADDOs capitalizes on the existing ADDO regulatory systems. Regulatory oversight and support by district- and ward-level inspectors help ensure that ADDO dispensers are held accountable for selling ACTs at the fixed price and for maintaining other ADDO standards. Supervision, focusing on education and mentoring of ADDO owners and dispensers, helps strengthen and maintain the quality of care and is vital to program sustainability. RPM Plus and TFDA trained and mentored council health management team supervisors at the district level to conduct quarterly supervisory visits with ADDO dispensers with the goal that the council team would eventually assume all supervisory responsibilities for ADDO dispensers, including their adherence to the ACT policy. Specifically, supervisors are supposed to ensure that ADDOs have ACT management tools on hand (monthly report forms, referral forms, etc.), provide technical support related to ACT dispensing, and evaluate dispensers' knowledge and skills. The main challenge with supervision is that the council health management teams also monitor public health facilities, but do not receive additional resources to cover transport costs and time needed to monitor ADDOs as well.

The use of ADDO drug registers to record all sales information helps monitor and quantify ACT sales. In addition, quarterly report forms were created for ADDO dispensers to collate information on the number and age of malaria patients treated and product losses due to drug expiry. ADDOs are required to submit these forms quarterly to the regional distributor.

### Training ADDO dispensers on ACT policy

Comprehensive training of both dispensers and owners is already a key requirement for the ADDO accreditation process. In addition to the six-week ADDO dispenser training program that had already taken place in Morogoro and Ruvuma regions prior to the ACT pilot program, RPM Plus and TFDA conducted an additional two-day training course for ADDO dispensers on the new malaria treatment policy. This training included the correct usage of ACTs, inventory management, and reporting on drug consumption, patient-related data, and ADRs. A total of 1,363 ADDO dispensers from 10 districts attended the training.

### Monitoring drug safety

As the availability of ACTs increases in the private sector through ADDOs, it is important to ensure that systems are in place to monitor and promote safe and appropriate use. TFDA already has an established passive (voluntary) ADR reporting system for medicines dispensed in public health facilities. To facilitate reporting by ADDOs, TFDA approved a simplified ADR reporting form for ACTs, inclusion of ADR reporting in ADDO dispenser training, and integration of ADDOs' ADR data into the district ADR reporting process. RPM Plus worked with the TFDA and council health management teams to link ADDOs with this reporting system. The ADDO dispenser training on ACT policy change addressed ADR issues to increase dispensers' awareness of the importance of drug safety monitoring and to instruct them on proper reporting using ADR forms. Dispensers were also instructed and encouraged on how to discuss drug safety issues with their clients. At the time of this evaluation, 20 ADDOs had reported on customers' "minor complaints" (skin rash and upset stomach) to the district authorities. Although these were nonspecific and might or might not have been related to taking ACTs, these early results indicated an increased awareness of the ADR issue.

### Behavior change and communication

Behavior change and communication (BCC) activities were deemed a critical part of this intervention. During the assessment and planning stages of the pilot, RPM Plus consulted with NMCP to map out BCC activities related to malaria and to link this pilot program to ongoing BCC work to leverage resources and ensure that a consistent message was being delivered on ACT policy.

The Communication and Malaria Initiative in Tanzania (COMMIT), which is led by the Johns Hopkins University's Center for Communication Programs, expanded their existing BCC activities related to the ACT policy in Morogoro and Ruvuma regions to include the availability of ACTs in ADDOs. COMMIT incorporated the availability of ACTs in ADDOs in road shows in the two regions and planned further strategies, including radio broadcasting and the dissemination of printed materials such as stickers, flyers, and t-shirts. However, a delay in the COMMIT BCC activities related to ADDOs meant that the activities did not coincide with start of ACT distribution, as planned, but instead started nine months later.

Additionally, the Ifakara Health and Research Development Center's ACCESS program, which works on malaria projects in two Morogoro districts (Kilombero and Ulanga), has been using a BCC and social marketing approach that promotes ACTs as the treatment of choice for malaria symptoms, refers patients to health centers to obtain treatment, and emphasizes completing the full dosage for effective treatment [14].

### Program evaluation

Approximately one year after the introduction of subsidized ALu in ADDOs, we planned this evaluation of the pilot program. One year allowed enough time to account for fluctuation in ACT consumption due to malaria seasonal patterns and to identify program barriers and identify recommendations to guide future delivery of subsidized ACTs in Tanzania's private sector. This program evaluation focused on processes in designing private sector subsidized ACT delivery, assessing the quantities of antimalarials that ADDOs dispensed, and the efficacy of regulatory and reporting systems related to ACTs. In addition, the evaluation sought to document challenges and lesson learned in launching an ACT delivery initiative through the ADDO program.

### Uptake and availability of ACT in ADDOs

One of the major advantages of the ADDO program is the improvement in record keeping, which is an accreditation requirement. Owners are required to keep records of all purchases and dispensing transactions for the list of products authorized for sale. To overcome logistical challenges and reduce the costs associated with collecting data from each individual ADDO, we asked ADDO dispensers to bring their drug registers, ACT purchase reports, and stock cards to central locations close to their communities. Most locations (primarily primary schools or ward offices) were in peri-urban areas with a few in villages. ADDO dispensers spent half a day in the meeting and were able to travel back to their shop. Prior to the meeting, the district team sent information such as which records to bring by calling or text messaging owners. The dispensers did not receive an allowance for participating, but were provided refreshments.

Four hundred forty-eight ADDOs from 10 districts participated in this data collection exercise, 298 in Morogoro region and 150 in Ruvuma region, representing nearly 70% of ADDOs in operation in the two regions. ADDO drug register data were available from July 2007-June 2008 for Morogoro region and from July 2007-September 2008 for Ruvuma region. The drug register review was used to extract data on the number and age of customers who were sold treatment for uncomplicated malaria for each month of the period, including the type of antimalarial dispensed. The evaluation team also reviewed ACT purchase reports that had been submitted to the regional distributors. Triangulation of various records allowed us to verify and crosscheck discrepancies and accuracy of quantities purchased and dispensed during any period. Additionally, a questionnaire was administered to the ADDO dispensers to determine what type of ACTs shops were stocking and the reasons why. We also asked dispensers about the availability of ACT tools in their shops, including quarterly report forms, standard operating procedures for ACT purchasing, price indicator sheets, and dosage indicator sheets.

### Study limitations

The data from the ADDO drug registers suffers from limitations that are common with routine records in developing countries; however, this intervention involved working with district teams and ADDO owners to strengthen supportive supervision and the quality of data recording related to ACTs in the ADDOs. Regular teams visited ADDOs and worked with owners to ensure completeness, accuracy, and timely reporting of ACTs dispensed.

The participation rate among eligible ADDOs was around 70%, which was an accomplishment given the logistical challenges (e.g., owners had to close the shop to attend). Follow-up with those who did not participate indicated that they did not receive the information about the evaluation workshop from the district health office, which was confirmed through government sources; however, other reasons may have contributed and may have biased the results. No particular geographic areas lacked participants; no-shows were fairly evenly distributed. In addition, problems still exist with ACT sales not being recorded in the drug register; however, observations from previous supervision visits suggests that this issue is not limited to the ACT program, but rather is related to overall underreporting of medicine sales to avoid taxes. This under-reporting, however, may bias overall results.

The distribution of subsidized ACTs though ADDOs was piloted in all existing ADDOs in the two regions of Morogoro and Ruvuma to provide information on feasibility and implementation before large-scale nationwide rollout. One of the key requirements was that the implementation needed to be designed as a routine activity to reflect the reality on the ground and to assess its feasibility rather than a strictly controlled design using a naive region. From routine supervision and monitoring visits, we had documented that no ACTs were available in ADDOs prior to the intervention. Because the price for unsubsidized Coartem had been USD 8-10, none of the ADDOs could afford to stock it because customers could not afford to pay.

## Results

### Uptake and availability of ACT in ADDOs

As mentioned, results are based on data from 448 ADDOs--or about 70% of the total number of ADDOs in the 10 target districts. Over the pilot program implementation period (July 2007-September 2008), the 448 ADDOs dispensed antimalarial treatment to over 300,000 customers. Around 30% of malaria treatments were for children under five. Table 2 presents district-specific data on ADDO patients.

**Table 2:** Number of customers dispensed with antimalarial medicines at ADDOs by district: July 2007-September 2008

Region	District	# ADDOs participating in ACT delivery	#ADDOs participating in ACT evaluation	# Clients seen at ADDOs (all conditions)	#Recorded clients dispensed with antimalarial (% of total clients)	# Recorded clients dispensed antimalarial who are children <5 (% of total clients dispensed with antimalarial)	Clients dispensed with ALu	Clients dispensed with SP	Clients dispensed with amodiaquine	Clients dispensed with quinine
	Mvomero	71	54	54,979	17,597 (32.0)	5,433 (30.9)	27%	41%	18%	14%
	Morogoro Rural	41	33	43,375	12,703 (29.3)	4,593 (36.2)	47%	29%	13%	11%
Morogoro	Ulanga	50	30	83,933	36,100 (43.0)	12,460 (34.5)	19%	54%	18%	9%
	Kilombero	126	67	231,021	63,902 (27.7)	28,588 (44.7)	10%	59%	11%	20%
	Kilosa	167	134	142,513	43,337 (30.4)	12, 411(28.6)	21%	60%	9%	10%
	**TOTAL**	**455**	**(298)66%**	**555,821**	**173,639 (31.2)**	**63,485(36.6)**	**19%**	**55%**	**12%**	**14%**

	Tunduru	25	9	40,980	8,480 (20.7)	3,387 (39.9)	11%	46%	16%	27%
	Mbinga	56	43	152,918	51,996 (34.0)	12,637 (24.3)	24%	47%	18%	11%
Ruvuma	Songea Rural	40	34	63,480	21,231 (33.4)	8,925 (42.0)	23%	38%	21%	18%
	Namtumbo	26	13	25,068	8,802 (35.1)	3,135 (35.6)	33%	35%	15%	17%
	Songea Urban	58	48	184,945	46,718 (25.3)	11,349 (24.3)	18%	58%	11%	13%
	**TOTAL**	**205**	**(150) 78%**	**467,391**	**137,227(29.4)**	**39,433 (28.7)**	**22%**	**48%**	**16%**	**14%**

The percentage of ADDOs that dispensed at least one course of ACT rose from 26.2% during July-September 2007 to 72.6% during April-June 2008. For the five districts in Ruvuma that reported data for July-September 2008, this increased to 81.5%. At the time of the evaluation, dispensers reported that 68% of ADDOs in Morogoro and 78% of ADDOs in Ruvuma were stocking at least one of the four types of ACTs (indicated by yellow, blue, and red, green packaging). The main reasons they cited for ADDOs not stocking ACTs included the far distance to the regional distributor (92%), the high sales price of ACTs (55%), and the ADDOs' limited capital resources (52%). Slow uptake in ADDOs (as reported by dispensers) was also associated with patient perceptions that the ACT dosing schedule was difficult (30%) and the availability of free ACTs at public health facilities (10%). Only 4% of dispensers cited unavailability of ACTs at the regional distributor as a reason for not stocking. Dispensers also demonstrated a high level of compliance with ACT documentation requirements; over 80% of ADDO dispensers reported using drug registers, ACT quarterly report forms, ACT price indicator sheets, ACT dose indicator sheets, and referral forms. Evaluation teams verified all the records presented by ADDO dispensers.

**Figure 4:**
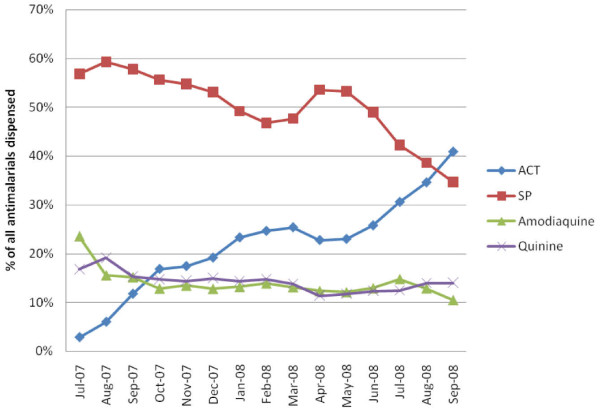
**Percentage of customers dispensed antimalarials in ADDOs by month: July 2007-September 2008**.

The ADDO data indicated that the number of customers seeking malaria treatment with ACTs gradually increased after the start of the pilot program, while the use of non-ACT antimalarials declined (Figure 4). In the 448 ADDOs reporting, ACTs went from 3% of all antimalarials sold in July 2007 to 26% in June 2008. In the five districts of Ruvuma where data was available up to September 2008, ACTs comprised 41% of all antimalarial sales in September 2008. Concurrently, sales of the most common antimalarial, SP, comprised 57% of all antimalarials distributed in July 2007, decreasing to 49% in June 2008, and further to 35% in September 2008 (for the five Ruvuma districts). Small decreases in amodiaquine and quinine sales were also evident over the intervention period.

**Figure 5:**
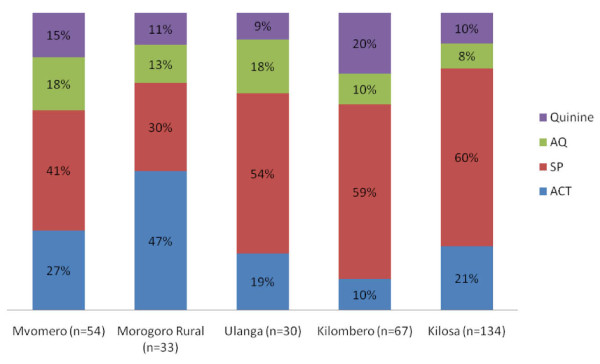
**Proportion of antimalarial medicines dispensed at ADDOs by district in Morogoro, July 2007-September 2008**.

District-specific data showed substantial variation among the districts in ACT uptake through ADDOs, ranging from ACTs representing 10% of all antimalarial sales in Kilombero to 47% in Morogoro Rural (Figure 5).

## Discussion

This paper attempts to provide policy makers and program managers with concrete results from a year-long pilot program to guide their policy, regulatory, and implementation decisions for scaling up private sector delivery of ACTs through ADDOs. Although ADDOs have been operating in Tanzania on a limited scale since 2003, the use of subsidies in the private sector to promote the sales of a particular treatment--in this case, ACTs, is new; therefore, this data is an important contribution to the government's decisions in relation to developing a strategy to increase access to ACTs.

As the results of this evaluation show, with extensive collaboration by numerous stakeholders a sustainable and reliable system to increase access to ACTs through the private sector has been established in two regions of Tanzania, using legally recognized accredited drug shops, the existing private sector pharmaceutical distribution system, trained drug dispensers, and local supervisory and regulatory oversight. Additional steps in program implementation involved procuring subsidized ACT products, determining packaging and pricing, training dispensers in the new ACT policy, and establishing an ADR monitoring system. Preliminary results showed that the intervention increased access to affordable ACTs for rural and underserved populations. In addition, early signs showed that antimalarial monotherapies not recommended by NMCP are being "crowded out" of the market. Importantly, this transition has been accomplished in an environment where the safety of the drugs and the quality of services are being monitored and regulated. While the results of the pilot program evaluation are encouraging, we have identified a number of challenges and opportunities for improvement.

### Distribution chain

One year after initiation of the pilot, 70% of ADDOs that had reported data (which had represented about 70% of the total number of ADDOs in the two regions) for this pilot program were stocking ACT products. This figure exceeded 75% in 6 of the 10 districts. In the other four districts (Kilombero, Namtumbo, Tunduru, and Ulanga), the primary reason dispensers said that ADDOs did not stock ACTs was the long travel distance to the regional distributor for re-supply. This suggests that the current distribution system for ACT products, based on having one regional distributor in each region, is inadequate to meet the needs of ADDOs with limited capital or remote locations. For example, in Tunduru district, only 35% of ADDOs stocked ACTs at the time of data collection, and 80% of those dispensers cited distance to the distributor as the reason. In addition, a number of ADDOs reported having inadequate capital to procure enough ACTs to last until their next purchase, which is affected by long distances and poor road conditions.

The best solution for distribution chain problems is to ensure that more certified wholesalers at the district level are available to serve ADDOs. Establishing certified wholesalers is a relatively slow and difficult process; for example, TFDA requires ADDO restricted wholesalers are to be supervised by a pharmacist, who is seldom available in rural areas. An interim option is to revisit the current requirement and consider allowing lower pharmaceutical cadres to supervise select ADDOs to serve as an ACT distributor to more remote ADDOs. Both strategies would help ensure that ADDO owners have a shorter distance to travel to obtain ACTs, lower travel costs, and greater ability to refill their stock. However, this option has regulatory implications that TFDA needs to consider carefully.

### Sales factors

While the pilot program demonstrated a generally successful mechanism to increase the availability of ACTs in the private sector, the sales of ACT products comprised only 26% of overall antimalarial sales in the 10 districts. The two main reasons cited by ADDO dispensers for the poorer than expected sales included the high prices of ACTs and the patient's perceptions of the drug. In addition, although our results show an increase in ACT sales at ADDOs, we cannot determine if this increase indicates an overall increase in ACT use in the community or merely a shift in access from one ACT source to ADDOs.

Two studies have examined Tanzanian caregivers' willingness and ability to pay for ACTs for a sick child: one found a median level of willingness to pay of TSH 600 (USD 0.46) and the other found a median willingness to pay of TSH 650 (USD 0.5) [12,15]. This suggests that the fixed price of TSH 500 (USD 0.38) for the child's course of ACT in ADDOs is appropriate. We did not identify any studies examining willingness to pay for an adult course of ACT; however, the study by Wiseman et al. found that 95% of caregivers were unwilling to pay TSH 1,500 (USD 1.15) (cost of the adult course of ACT in ADDOs) for a child's course of ACT [12]. Presumably, a high percentage of people might also be unwilling or unable to pay TSH 1,500 for an adult treatment. As marketplace prices of SP for adults at the ADDO level range from TSH 300-1000 (USD 0.23-0.77), depending on the brand and packaging (loose pills versus blister pack), one adult course of ACT could cost the same as up to five treatments with SP.

The subsidized ACTs currently available in Tanzania have a more complicated dosing schedule than other common antimalarials, requiring two doses per day for three days versus a single dose for SP. Depending on the patient's age, one treatment comprises between 6- 24 pills. It is not known to what extent the relative inconvenience of the dosing schedule or other factors related to the appearance of the drugs or the packaging deters individuals from purchasing ACTs in ADDOs. Patient compliance to the proper ACT regimen purchased from ADDOs is also unknown. Further research examining the social, economic, and cultural factors affecting malaria treatment decision making would contribute to the design of effective BCC messages and marketing strategies for the new ACT policy.

During the pilot program monitoring, supervisors noted that the profit margin for ADDO business owners for the adult course of therapy of subsidized ACT was significantly less (28-44%) than that for SP (61-164%) (See table 3). Thus, some owners may have been reluctant to recommend ACTs to treat malaria and risk losing profits. In March 2009, after reviewing the prices of ACTs, in consultation with NMCP and TFDA, we decided to decrease the wholesale and retail prices of all ACT age categories from TSH 350-1,250 to TSH 200-700 (wholesale) and from TSH 500-1,500 to TSH 300-1,200 (retail). The new retail price is about USD 0.23-0.92. This put the profit margin for ACTs in line with that of SP (an average of 71%) and may encourage more dispensers to recommend ACTs to their clients.

**Table 3:** Comparison of Antimalarials Price and Percent Mark Up at ADDO

Product	Wholesale Price Tsh [$]	Retail Price at ADDO Tsh [$]	Profit Amount Tsh [$]	% Profit Margin
**SP**	180-500 [0.14-0.38]	500-800 [0.38-0.62]	300-320 [0.23-0.25]	61-164%
**Amodiaquine**	200-500 [0.15-0.38]	500-800 [0.38-0.62]	300 [0.23]	61-153%
**ACT**	350-1250 [0.27-0.96]	500-1500 [0.38-1.15]	150-350 [0.12-0.27]	28-44%
**Quinine**	85 [0.065]	100 [0.076]	15 [0.012]	19%

Before the start of the subsidy program, other studies had documented no or very low availability of ACTs in the private sector, including ADDOs [16]. This intervention provided the first subsidized ACTs to the private sector market at a price deemed affordable for the majority of the population living in rural areas. ADDO dispensers either provide ACTs after they receive prescriptions from health facilities (assuming proper diagnosis was done there) or they make a decision to dispense ACTs for cases of uncomplicated malaria based on their training and experience. Currently, ADDO dispensers are not allowed to use a test to diagnose malaria.

The overall uptake of ACTs in ADDOs was clearly undermined by the sales of alternative antimalarials, particularly SP. Once Tanzania establishes an adequate ACT supply for the country, it is expected that TFDA and NMCP will remove SP and other suboptimal antimalarials from ADDOs. This should limit the availability of other antimalarials in the market and increase the use of ACTs. Discussions continue about whether SP and other less effective antimalarials should be removed only after nationwide coverage of subsidized ACTs in ADDOs has been achieved, or whether the removal policy can be phased in as regions begin the program.

### BCC strategy

The lack of a comprehensive BCC strategy specifically targeting ACT availability in ADDOs is a significant barrier to encouraging communities to purchase ACTs in ADDOs to treat uncomplicated malaria. A small pilot program to distribute subsidized ACTs through DLDB, carried out by the Clinton Health Access Initiative (CHAI) in two districts of Tanzania, observed more rapid uptake of ACTs through DLDB than we observed in this study. One reason cited in the CHAI study was a more extensive and focused social marketing campaign that involved advertising the availability of ACTs in drug shops through numerous media outlets [17]. The COMMIT project initiated BCC activities to improve the use of ACTs in the community and to promote the availability of subsidized ACTs through ADDOs (Personal communication, Waziri Nyoni, March 2009). COMMIT plans to conduct radio spots and distribute fliers and posters in the regions that have ADDOs. These efforts should help disseminate information to communities about the availability of ACTs in public health facilities and ADDOs and to encourage the use of ACTs for uncomplicated malaria.

### Dispenser training

The importance of drug seller training is well recognized and has been a vital component of most interventions aimed at improving the quality of services provided by drug shops and other medicine sellers [4]; however, the comprehensive nature of the six-week ADDO training program is unique from other medicine seller interventions, which are generally more narrowly focused. For example, many of the dispensers in the CHAI program to distribute ACTs through DLDB had no formal medical training and only attended two-day training on malaria and ACTs [17]. In contrast, prior to the two-day ACT training for this pilot program, ADDO dispensers already had a minimum of one year of health training as a nurse assistant and had undergone a six-week course in general dispensing, pharmaceutical management, and integrated management of childhood illness. Their ADDO training covered how to properly identify and treat malaria, recognize danger signs of complicated malaria, refer patients to health facilities, and properly keep records and comply with regulations governing proper dispensing practices. While our evaluation did not specifically assess ACT dispensing services in ADDOs, evidence indicates that the quality of dispensing services in ADDOs is high, making it more likely that ADDO dispensers dispense ACTs responsibly [18]. In regions where the ADDO program has yet to be initiated, the basic dispenser training course will include the new ACT guidelines, inventory control practices, and reporting, which will eliminate the need for a special ACT-specific training course.

### Regulatory and supervisory support

The regulatory and supervisory systems inherent in the ADDO program and then adapted to monitor ACT dispensing make this intervention stand apart from other interventions to provide subsidized ACTs in private drug shops. While implementing these systems seems slow and requires significant financial and human resources, they help ensure that the population receives quality products and services and that public health goals are met.

NMCP had initially raised ACT leakage as a concern before the program began; however, during the pilot program, no leakage of ACTs destined for the public sector to the ADDOs was identified. To prevent leakage from the public sector to ADDOs or from ADDOs to DLDB, a number of safeguards were put in place: (1) ACTs meant for ADDOs included a special ADDO sticker (figure 2); (2) ADDO owners had to present their accreditation certificate to the regional wholesaler to purchase ACTs; (3) teams monitored sales and reviewed stock records monthly at the national and regional suppliers; and (4) district- and ward-level inspectors checked shops for public-sector ACTs (and found none).

Another concern was the potential risk associated with indiscriminant use of the drugs, which can accelerate the development of drug resistance and increase the risk of ADRs [4]. This pilot program addressed these concerns by training dispensers, implementing supervisory and regulatory systems, and linking ADDOs with an ADR reporting system.

At the time of writing this paper Tanzania was among the countries selected to implement the Affordable Medicines Facility-malaria (AMFm). The AMFm, a price subsidy mechanism to expand access to affordable ACTs, is managed by the Global Fund to Fight AIDS, Tuberculosis and Malaria and has been described elsewhere [19,20]. The planned AMFm strategy in Tanzania has implications for the ADDO program. Currently, in the private sector only Part 1 pharmacies and ADDOs can legally stock and dispense ACTs, which makes it difficult to increase access to ACTs in regions without ADDOs. TFDA acknowledges the challenges in scaling up the ADDO program nationwide in a reasonable time frame. However, TFDA is concerned with an approach that essentially favors quick results by granting unaccredited shops additional dispensing responsibilities without full accreditation. Disregarding the ADDO program's comprehensive system improvements creates the danger of obstructing the nationwide ADDO implementation goal. TFDA does not want to send a mixed message by allowing both DLDB and ADDOs to deliver subsidized ACTs, especially if the DLDB have not started the accreditation process. Such an allowance may create a disincentive for unaccredited shop owners to participate in the ADDO program, which damages the program's chances for sustainability.

This evaluation found that the availability of management tools related to ACT dispensing in ADDOs was high. But while the majority of ADDOs had drug registers, ACT quarterly report forms, ACT price and dose indicator sheets, and referral forms, the evaluation suggested the need to improve dispensers' use of these tools. For instance, comparing the sales of ACTs recorded in drug registers with the purchasing slips from regional distributors indicated that dispensers were only recording approximately 60% of ACT sales. In addition, up to 70% of all malaria medicines sold in ADDOs are in response to written prescriptions from health facilities presented by patients, but in many instances, the sales of ACT from these prescriptions were not recorded in the drug registers. Using SP as an example, appropriate record keeping could indicate the percentage of SP sales that resulted from appropriate prescriptions to pregnant women and what percentage of sales was inappropriate. Strengthening supervision to ADDOs and emphasizing the importance of record keeping will continue to be important to ADDO program sustainability.

## Conclusions

As Tanzania expands access to effective antimalarial medicines, particularly in rural areas, ADDOs provide the mechanism to increase access in the private sector while ensuring quality of services and products to safeguard the public's health. This pilot program offered critical lessons and identified key challenges that need to be addressed for a successful and sustainable national scale-up. Market forces (price) alone will not increase access. Specific policy recommendations to assure the success of this strategy include: expanding the distribution system by allowing selected ADDOs in remote locations to resell products to other ADDOs until enough ADDO-restricted wholesalers are available to meet ACT as well as other ADDO product source needs; developing a comprehensive BCC strategy targeting ACTs in ADDOs; and developing policies that provide the districts with the capacity to supervise and inspect ADDOs to ensure quality of dispensing services and record keeping for accountability and monitoring. In addition, TFDA needs to continue to address the issue of under-reporting of sales of prescription-only medicines in the ADDO drug registers. TFDA should also explore opportunities offered by new technologies, such as use of cell phones in data collection and transmission.

ADDO owners' participation in the pilot, the first of its kind to deliver subsidized products in Tanzania, was critical. The program provided incentives in terms of an adequate profit margin to cover owner operational costs and small profit. But a majority of ADDO owners and dispensers participated because they felt this program enhanced their reputation in the community.

An intervention such as the ADDO program provides a platform to integrate interventions such as increasing access to ACT; however, such program requires substantial human and financial resources, high stakeholder participation, and time. Other countries planning to use the private sector drug sellers to increase access to ACTs can use such interventions as an initial step toward more comprehensively addressing the overall performance of the private sector health system. A drug seller intervention of limited scope can introduce ACT distribution with minimal dispenser training; however, the right path for a broader and more sustainable program should be based on innovative uses of the existing regulatory system, policy change, business incentives, capacity building, product quality monitoring, and consumer awareness and education.

## Competing interests

The authors declare they have no competing interests other than employment that includes activities related to the ADDO program.

## Authors' contributions

ER substantially contributed to the project design, data analysis and interpretation, and drafting and revision of the manuscript. BM helped analyze and interpret the data and drafted the early manuscript. JL collected the data, participated in data analysis and interpretation, and helped draft the manuscript. RM participated in data collection and analysis and drafting of the manuscript. WM participated in the design and implementation of the project and data collection and analysis. ME critically reviewed and revised the manuscript and contributed intellectual content. MG helped draft and review the manuscript. ES and BK participated in project design, data collection coordination, and helped draft the manuscript. SK participated in data collection and interpretation and helped draft the manuscript. All authors read and approved the final manuscript.
